# miReader: Discovering Novel miRNAs in Species without Sequenced Genome

**DOI:** 10.1371/journal.pone.0066857

**Published:** 2013-06-21

**Authors:** Ashwani Jha, Ravi Shankar

**Affiliations:** Studio of Computational Biology & Bioinformatics, Biotechnology Division, CSIR-Institute of Himalayan Bioresource Technology, Palampur, Himachal Pradesh, India; Vanderbilt University Medical Center, United States of America

## Abstract

Along with computational approaches, NGS led technologies have caused a major impact upon the discoveries made in the area of miRNA biology, including novel miRNAs identification. However, to this date all microRNA discovery tools compulsorily depend upon the availability of reference or genomic sequences. Here, for the first time a novel approach, miReader, has been introduced which could discover novel miRNAs without any dependence upon genomic/reference sequences. The approach used NGS read data to build highly accurate miRNA models, molded through a Multi-boosting algorithm with Best-First Tree as its base classifier. It was comprehensively tested over large amount of experimental data from wide range of species including human, plants, nematode, zebrafish and fruit fly, performing consistently with >90% accuracy. Using the same tool over Illumina read data for *Miscanthus,* a plant whose genome is not sequenced; the study reported 21 novel mature miRNA duplex candidates. Considering the fact that miRNA discovery requires handling of high throughput data, the entire approach has been implemented in a standalone parallel architecture. This work is expected to cause a positive impact over the area of miRNA discovery in majority of species, where genomic sequence availability would not be a compulsion any more.

## Introduction

miRNAs are ultra-short RNA molecules with an average length of ∼21 bases [1], [2]. Mature miRNAs are formed through a series of post-transcriptional processing steps [3], [4]. The canonical pathway of miRNA biogenesis involves cleaving of pri-miRNA by a nuclear RNAse III enzyme complex, Drosha-DGCR8, into a shorter precursor form, pre-miRNA. This processing step removes the unpaired terminal tails. The pre-miRNA is transferred to the cytoplasm, where it is cleaved by Dicer RNAse III enzyme, releasing an ultra-short double stranded fragment [5]. One of these strands acts as a mature miRNA, while the complementary/partially complementary partner strand is called as star (*) strand. However, there have been several instances where the star strand has been found acting as a mature miRNA, targeting mRNAs. It is expected that miRNAs regulate more than 1/3rd of genes [6] and exist as a prime component of eukaryotic regulatory systems.

Presently, miRBase [7] has a total of 21,264 reported miRNAs which belong to a total of 1,133 different families and 193 different species. Still, a lot of miRNAs are to be identified and characterized. Initially, experimental methods like those based on PCR and blotting approaches were used extensively to identify mature miRNAs [1], [8]. Computationally, majority of these miRNAs have been characterized using some set of rules to identify precursors, relying largely upon existence of hairpin-loop structure, homology and thermodynamics. Recently, some machine learning based approaches claimed good success in identifying precursor miRNAs [9]–[12]. However, there is a limited number for tools to identify mature miRNAs. Tools like MatureBayes [13], MaturePred [14], MirPara [9] and miRRim [15] belong to this genre, and all of them essentially require a precursor/genomic sequence to identify mature miRNA regions. Some tools and algorithms use NGS read data for more accurate miRNA discovery. Such tools are miR-BAG [12], miRDeep [16], miRTrap [17], miRNAKey [18] and miRExpress [19]. The underlying principle of all these tools has been based on mapping of reads obtained from small RNA-seq sequencing to the genomic sequences of the concerned species. The mapped genomic regions are expanded further to accommodate a possible precursor miRNA region, which in turn is evaluated for its potentiality for being a precursor miRNAs through precursor identification algorithms.

Regardless of the way, whether the miRNAs are characterized using NGS read data or independent of it, there lays a uniform clause with all of them: All currently available miRNA discovery tools essentially require a reference/genomic sequence to identify a novel miRNA. Also, most of them are dependent upon identification of miRNA precursors. In such condition, non-availability of genomic sequences becomes a big limiting factor in the area of miRNA biology based research activities, including miRNA discovery. This has resulted into a sort of knowledge skew where most of the miRNAs have been reported only for those species whose genomic sequences are available or homologous sequences are known. Only 16% of total reported miRNAs in miRBase are from species whose genome is not sequenced. Also, the majority of these 16% miRNAs has been identified using homology search and exhibit a very few species specific miRNAs. Thus, barring a few model organisms, there is almost negligible miRNA information for most of the species (**Supporting Material S1**). Recently, a group suggested an approach to look for novel mature miRNAs from NGS read data, using some set of rules [20]. The authors considered only those reads as miRNA candidate which could form duplex, maximum four mismatches existed, 3′ overhangs were observed, high agreement existed across all reads for 5′ end, and duplex length stayed within the range of 18–24 bp. However, such rules have limitations and require validation over large amount of datasets. Based on PCR experiments over total 24 instances, the authors reported ∼40% false discovery rate. Validation could be obtained for only 13 instances. Also, the study did not report any comprehensive testing against known instances datasets in order to establish their approach.

Therefore, there is an immediate requirement to fill in such void which has an impeding impact over the area of miRNA biology in absence of any tool to identify miRNAs independent of availability of genomic sequences. Considering this, in the present work a novel approach and software has been introduced to identify mature miRNAs directly from NGS read data without any dependence upon genomic sequences or homologous references. Also, with this, cheaper runs of small RNA sequencing on NGS platforms like Illumina would be sufficient for mature miRNA discovery, facilitating miRNA biology and regulatory system understanding of most of non-model organisms whose genomic sequences are still not available.

## Methods

### Reads Assembling, Encoding and Representation as Instances

In the present system, the objective was to develop an approach which could identify mature miRNAs directly from the read data without depending upon genomic or homologous sequences. In this direction, the primary step was to develop a protocol for short small RNA read *de-novo* assembling, which could look across the connected reads, merge them together to form a single unique continuous stretch (contig). Since mature miRNAs usually exist in a duplex form with an inexactly pairing complementary star (*) strand, it becomes imperative to look for complementary contigs to develop the duplexes. Such duplexes form the training and testing instances or queries for the tool presented here.

The step of reads assembling was done considering two different conditions: (A) For model building (training and developing the classification system) and (B) For testing and *de novo* identification from small RNA sequencing read data. In case of model building and developing a classification system, the reads mapping to a particular miRNA (positive instance) or non-miRNAs (negative instances) were merged together for overlapping regions, yielding assembled sequences from the reads. However, in case of *de novo* identification, references are not available and the reads need to be searched among themselves for overlaps. Using Knuth-Morris-Pratt (KMP) algorithm, the reads were searched for terminal overlap with at least 5 bp overlap and without any mismatch. All such overlapping reads were merged to get the corresponding contig. The effectiveness of this assembling approach was tested for known miRNAs and their associated reads. All reads combined perfectly with right partners, suggesting a rare chance of mis-assembly.

#### Duplex formation and encoding

Next to the assembly step was identification of most suitable complementary partners. An in-house developed global alignment script with gap opening penalty of −5, gap extension penalty of −2 and with G:U pairing consideration was run to align the assembled contig sequences to each other for highest possible complementarity. Considering existence of terminal overhangs in mature miRNA duplexes, global alignment between the contigs was performed with scope for 3′ overhangs with two to zero nucleotide(s). The highest scoring pairs were considered as duplexes. All such duplexes were encoded into single sequence representation for four states: match, mismatch, insertion and deletion. The encoded duplexes made the final input instances to the classification system. Before going for classification or model building, every such encoded instance was transformed into a long range discontinuous single order transition probability vector. Sets of encoded pattern mature miRNA single order transition matrix profiles were formed for every possible positional pairings. The encoded patterns of miRNAs with same length were considered into a single common cluster, where encoded patterns were multiple aligned to form encoded pattern profile matrices. Every such matrix had five different sub-matrices, representing five possible pairing conditions’ transition probabilities for different positions in any given duplex. These matrices were used exclusively to derive the corresponding match states probabilistic representation of the encoded query sequence for classification. More details are given below.

### Duplex Transition Matrix for Features and Training

It has been reported that in a given precursor sequence, the mature miRNA region exhibits a high degree of conservation where interaction between the nucleotides of this region could be important [1], [21], [22]. A few recent works have reported binding sites for transcription factors and RNA binding proteins in the mature miRNA region of precursors. Presence of such sites might be causing regulation of miRNA transcription as well as post-transcriptional fates of miRNAs [23]–[27]. Such functionally important regions are usually conserved. Considering this, it was assumed that some sort of structural conservation existed for this region in miRNAs. Using mature miRNAs and their corresponding star (*) miRNA sequences from opposite strands, complementary alignments were formed, which were converted into single sequence representation for the four states of pairing: Match (M), mismatch (m), Insertion (I) and Deletion (D). By doing so, the structural states of miRNA regions were encoded, irrespective of nucleotide sequence underlying them. In this manner, the encoded structural patterns for all known miRNAs were developed for the target species in the present work. These patterns were clustered according to their length. For each of these clusters, their corresponding matching state transition profile matrices were built, having five different subprofiles: 1) profile for co-occurrence of mismatches 2) profile representing co-occurrence of mismatch and insertion 3) profile representing occurrence of mismatch and relative occurrence of deletion 4) profile representing co-occurrence of insertions, and 5) profiles representing co-occurrence of deletions. All profiles contained single order transition probability values for long distance transitions of states. The length of the matrix was the limiting size for the maximum possible distance. Each microRNA was scored against these matrices. Same was done for negative instances coming from the small RNA sequencing read data. The profile which returned the highest cumulative score for every transitional position score was considered as the representative profile, and the encoded pattern was transformed into an array of probabilistic scores, which acted as an n-features based input for classification steps. The *n*-features are the position specific transition probability values for the observed match states in the query. The Best-First Tree (BF-Tree) algorithm [28] was used as the base classifier, which primarily encounters the *n*-features inputs.

The number of such input attributes varied across the clusters due to variation in the length of matrices. In the present study, the single order transition between the various match states of miRNA duplex defined the attribute nodes (the *n*-features). The leaf nodes define the binary classes, i.e. instances are classified as a mature miRNA or else. A BF-Tree is an advanced form of decision tree, where multiple trees are considered to build a final tree. While building a BF-Tree, the best attributes are considered as the decision nodes in preference, performing binary split at every node for purity gain. The purity employs the degree to which instances of a particular class accumulate at a given decision node. A purity value of “1” indicates that no more decision nodes are required for extension, and the leaf node containing the class is attached to the specific node, reaching the purity threshold. Gini index was used as a measure of node purity. In the present study, post pruning was used with 5-fold cross validation. Preferential selection of best nodes and multiple hypotheses paths searches followed by cross-validation pruning to get the shortest tree, ensured reduction of over fitting and errors.

### MultiBoosting Implementation

In the present work, a highly reliable ensemble machine learning protocol, Multi-boosting, has been implemented. Multi-boosting [29] is Boosting with Wagging (a form of bagging), where for a given training data pool, random selection with replacement is done for training the base classifier. Every test instance is labeled with respect to the classifier for accuracy, based on which relative weighting of classifiers and instances is done. Every next step is dependent upon the previous step of learning, which also updates the weights in a manner where the most difficult instances to learn are weighted the most. Based on instance weighting, continuous sampling of learning data is done to train the newer classifiers. Unlike bagging, boosting does not give equal weights to all instances and classifiers but weights them according to their difficulty level and overall accuracy performance. Multi-boosting combines boosting and wagging by repeatedly running boosting for short sequences of training, then resetting the instance weights using wagging. The result is a committee of classifiers that consists of several subcommittees, each formed by boosting a wagged sample of the data. While designing any machine learning approach, bias and variance are two major factors to determine the performance ability of any classification system. It was found that compared to bagging based ensemble approach, boosting based ensemble approaches provide both: control of bias and variance. However, compared to boosting, bagging has better variance reduction. Therefore, Multi-boosting brings the strengths of bagging and boosting together, by avoiding over-fitting and providing the best bias and variance reduction. Boosting provides increment in error minimization with enhanced accuracy while wagging reduces the fluctuations and brings consistency. It has been found that compared to any single classifier algorithm, such ensemble algorithm performs better and stays stable even for largely variable input instances. In Multi-boosting, the subcommittees are formed by boosting. Output votes of subcommittees are returned to the set classifier and the final classifier for the given training set is built with weighting system. The same is repeated for “K”-number of iterations and classifiers, which together build an ensemble classification system of high accuracy and stability. The class which obtains the highest additive weights for any given instan*c*e is finally assigned as the class to which the instance is classified finally. A prominent property of Multi-boosting is due to wagging, where unlike bagging, the training data is repeatedly sampled from master data pool through a continuous Poisson distribution. The details of the Multi-boosting algorithm are given below:

### The Multi-boosting Algorithm

**Input:**

D, a set of *d* class labeled training tuples.

K, the number of iterations//each generating one classifier.

BL, base learning algorithm//BF-Tree here.

I_i_, *Vector of integers specifying the iterations at which each subcommittee i > = 1 should terminate.*

*1 Set i = 1;*

*2 for t = 1 to K do//for each classifier.*

3 *I_i_ = t, then*

4     *reset D*′ *to random weights from continuous Poisson distribution*

5    *normalize D*′ *to n*

6     *increment i.*

7     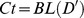


8     *Compute model error e(Ct), the error rate for training sample D*′, *where*

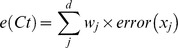



9     *if model error e(Ct) >0.5:*

10      *reset D*′ *to random weights drawn from continuous Poisson distribution*

11      *normalize D*′ *to n*

12      *increment i*

13      *got to step 7*

14    *Otherwise, if e(C_t_) = 0:*

15      *Weight of the classifier in voting, 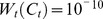
*

16      *reset D′*

17      *increment i*

18    *Otherwise:*

19      
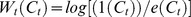


20      *for each training instance X_j_ ∈ D′*

21       *Multiply each misclassified training instance weight W_j_ by 1/2e(C_t_)*

22       *otherwise,*

23        *multiply W_j_ by 1/2(1-e(C_t_)*

Output:


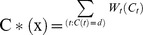
//assign the class which obtains the highest total weight.

The base classifier is the basic learning algorithm on which boosting works to refine the performance further by handling the hard to learn instances. In the present study, the above mentioned BF-Tree was implemented as the base learner. As mentioned above, the size of input attribute list to this tree varies according the profile selected to model the miRNA candidate. The maximum number of iterations steps taken was 100 while minimum 30 iterations were taken.

### Overview of miReader Working Algorithm

**1** Set of reads obtained from small RNA sequencing, **R** = {r_1_,r_2_,….,r_n_}.

**2** Initialize the set of pairing states based transition matrices, **M**, represented in *l*×5 dimensions, where, *l* is the length bound of the corresponding matrix.

**3** For each read **r** ***_i_*** **∈R**:

**4**    if (any suffix substring ***A*** of read **r** ***_i_*** i.e. **r** ***_i_***[1:x] matches another read’s prefix sub-string ***B,*** i.e., **r_j_**[y,l] OR **r_j_**[y,l] matches some read **r_j_**[1,x], where all matching substrings have length equal or higher than 5):

**5**    Report match, and merge ***A*** and ***B*** to form merged string S_i_*(intermediate).

**6**    update contig string **C_i_** = S_i_*.

**7**   repeat the same till the ***n^th^*** read.

**8**   populate the set of assembled contigs **C** = {C_1_,C_2_,…C_n_}.

**9** Search for complementarity between the contigs of set **C**:

**10**   Complementarity contig pair 
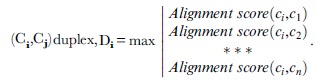



**11** Convert duplex *D_i_* into encoded pattern, ***p*** **_i_**, composed from alphabet of four match states |A| = {M,X,I,D}.

//where, M = complementary match, X = mismatch, I = insertion D = deletion.

**12** Scan ***p_i_*** against the set of 5-state transition matrices, **M**, to obtain the highest scoring matrix:

**13**   for each position ***“j”*** in pattern ***p_i_***:

**14**    determine match state, ***St_j_***, using alphabet |A|.

**15**    if (***St_j_*** ! = ‘M’):

**16**      Identify all other !M states (***St_j_***), for all previous positions, from ***i*** = 1 to ***j***-1.

**17**      Score for ***j*** **^th^** position, Score*_j_* = max(***St_i_*** ** ***→ St_j_***), where ***i<j.***

**18** Score a given matrix scan *n*, MatrixScore_n_ = 
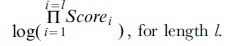


**19** Representative Score, R*score* = max(MatrixScore*_n_*), for all matrices of set **M**.

**20** Transform pattern ***p_i_*** into a vector of transition scores, ***V_i_***, for each position ***j*** *,* using Score***_j_***.

**21** Input ***V*** *_i_* to the Multiboost ensemble classifier C*.

**22** Class, **C** = max(C*( ***V_i_*** **)**)//return the class obtaining the highest composite weight from the ensemble system.

**Figure 1:**
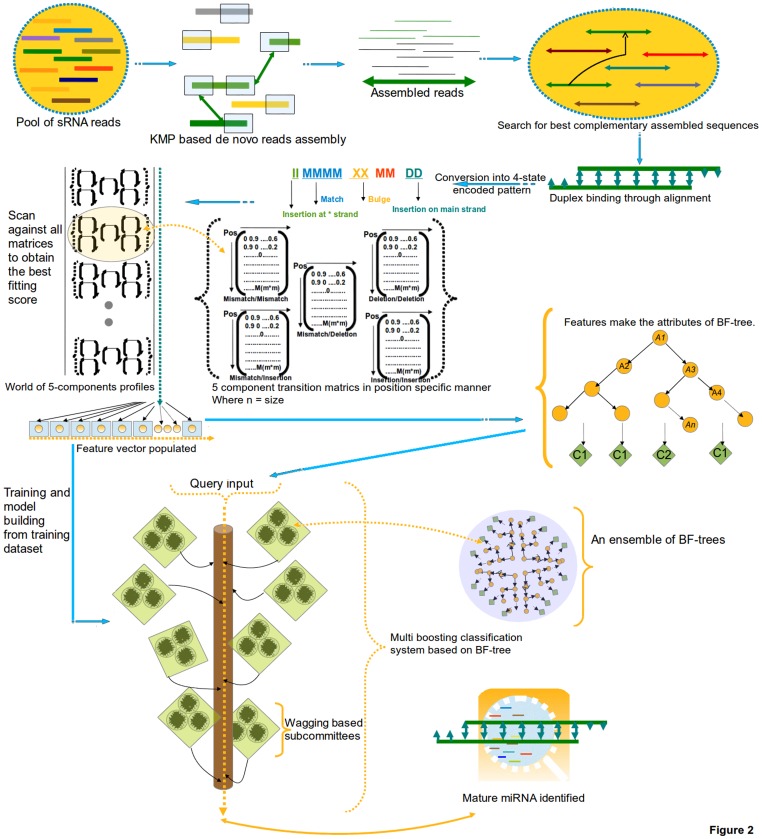
Work flow of miReader algorithm.

A thorough illustration of entire algorithm has been given in **Figure** **1**.

### Reads Data and Datasets Generation

Small RNA reads data which could map to known mature miRNAs were required to develop suitable models and training sets as well as testing sets. Small RNA reads data were downloaded from NCBI’s Gene Expression Ominbus (GEO) for seven different species viz. *C. elegans (GEO ID: GSE18634; Total reads: 151,017,180)*, *Drosophila(GEO ID: GSE7448; total reads: 2,075,098)*, *Arabidopsis(GEO ID: GSE26356; total reads: 12,756,632)*, *Oryza sativa(GSE38480; total reads: 17,913,317)*, human (GEO ID: GSE21722 with total reads: 425,505; GSE19812 with total reads 19,759,390), Zebrafish(GEO ID: GSE:27722; total reads: 29,963,921) and *Miscanthus giganteus(GEO ID: GSE28755, total reads: 12,400,937).*

In case of human, one set of small RNA read data was downloaded from GEO experiment series GSE21722. This contained small RNA reads derived from Roche 454 sequencing. Previously, a total of 221 human miRNAs were previously identified from this data, making it a suitable data source for training and testing set building. A total of 425,505 reads were pooled together and mapped on humam pre-miRNA sequences reported in miRBase version 19 [7]. This returned a total of 75,256 reads mapping across 919 human pre-miRNA sequences (out of 1,600 pre-miRNAs). A total of 644 pre-miRNAs were found with two or more reads mapping on them. Only such miRNAs and associated reads were considered for further studies. All such reads mapping to known pre-miRNAs were considered as positive instance components. A positive instance component is a read which maps to a known miRNA. Assembling of all such positive instances common to some miRNA leads to formation of a single stranded contig. Across the pool of contigs, the complementary contig pairs are searched to form potential miRNA duplexes using above mentioned protocol. Every such duplex representing a mature miRNA duplex has been considered as a positive instance, to which all corresponding component reads are linked and called positive instance components. Similarly, the small RNA reads mapping to non-miRNAs were considered as negative instance components. Detailed description about entire process of reads assembly and instance (encoded duplex) formation has already been elaborated in the above sections. In case of human, there were a total of 1,000 positive instances, a number higher than the total mapped pre-miRNAs (644). It was found that there were many pre-miRNAs for which two or more different duplexes formed, suggesting possibility for more than single mature miRNAs resulting from any single precursor. Around 500 assembled miRNA duplexes were taken as positives instances (miRNA duplexes obtained after read mapping and assembling) to build the training set. The remaining 500 positive instances were included in the testing set. For negative set creation, the reads were mapped on other classes of non-coding RNAs (i.e. tRNA, snRNA, snoRNA, rRNA), resulting into a total of 178,536 reads mapping across 3,966 non-miRNA non-coding RNAs. For 1,892 non-coding RNA sequences, two or more reads were found mapping. After assembling, the resultant negative set contigs were searched for complementary sequences to form duplexes which formed the final negative instances.

Another set of human small RNA reads were taken from an Illumina sequencing based experiment (GSE19812). The reads from Roche platform could map to only 644 miRNAs while more than 1,600 mature miRNAs have been reported in miRBase v19.0. Therefore, this was done to enrich the existing data and get a better representation of existing human miRNAs. A total of 1,265,903 reads mapped across 1,027 human pre-miRNAs reported in miRBase v19.0. After assembling, these reads produced a total of 7,893 unique assembled and non-overlapping sequence contigs. After complementary search, duplex could be formed for a total of 2,000 contigs, yielding a 1,000 positive instances (miRNA duplex candidates). The positive instances were distributed equally across the training and testing sets. A total of 69,335 unique reads (representing a total of 2,161,550 reads) mapped to other ncRNAs. A total of 7,193 unique assembled sequences resulting from the reads mapping to other non-coding RNAs were considered as negative instances. Similarly, the assembled contigs from reads mapping to miRNAs were considered as positive instances.

Similar protocol was followed for all target species considered in the present study for model generation viz. Human, *Drosophila*, *C elegans*, zebrafish, *Oryza sativa* and *Arabidopsis*. **Table 1** presents the detailed description on dataset generation for all species. The non-coding reference sequences were downloaded from Ensembl (www.ensembl.org). MiRNAs for all species were downloaded from miRBase version 19.0.

**Table 1:** Read data, training and testing set information for each species.

	*C elegans*	*Drosophila*	*Homo sapiens (Illumina)*	*Homo sapiens (Roche)*	*Arabidopsis*	*Oryza*	*Danio rerio*
Total reads	151,017,180	20,75,098	19,759,390	425,505	12,756,632	17,913,317	29,963,921
Total reads mapped on pre-miRNA sequences	48,991,955	929,257	1,265,903	75,256	896,956	2,110,263	872,363
Total unique reads mapped on pre-miRNAs	6,280	5,728	62,447	13,207	6,391	2,605	6,210
Total reads mapped on other ncRNAs	18,353,834	756,982	2,161,550	178,536	1,192,612	3,555,853	7,587,329
Total unique reads mapped on ncRNAs	63,599	67,183	69,209	18,362	13,836	28,559	14,579
Number of unique encoded positive duplexes (training)	97	100	500	500	100	250	143
Number of unique encoded positive duplexes (testing)	97	99	500	500	100	250	142
Number of unique encoded negative duplexes (training)	97	100	500	500	100	250	143
Number of unique encoded negative duplexes (testing)	97	99	500	500	100	250	142

Expression based analyses were performed using mapped reads count based approach, utilizing the below mentioned equation:

miRNA abundance (RPKM) = No. of reads mapped to the assembled sequences * 10^−9^/(total reads in the experiment).

Weka package was used to develop the classifiers. The entire software package, named as miReader, has been developed in standalone versions for Linux and Windows OS, using Java, Qt C++ GUI library and PERL.

## Results and Discussion

As already described above, most of the existing tools to identify miRNAs are mainly pre-miRNA identification tools, which essentially require genomic sequences. Compared to them, very few tools exist to identify the mature miRNA duplexes. Ironically, all these tools also depend upon the availability of genomic sequences, as they require derivation of precursor miRNA secondary structure as their first step. Tools like MatureBayes [13] and MaturePred [14] find the mature miRNA duplexes in a given pre-miRNA hairpin-loop structure. Both the tools fold the candidate genomic sequence into secondary pre-miRNA structure and index all nucleotides alongwith their corresponding pairing states (Match and Mismatch), considering a total of eight combinations of nucleotides and pairing states. Values for these combinations are estimated for the probable miRNA duplex region as well its flanking regions. It also considers the relative distance from the terminal loop of the precursor, duplex free energy as well as distribution of triplet structures for the duplex region. The last property was introduced by Xue *et al* [10] for identification of pre-miRNAs. Earlier, authors of MatureBayes had also reported pairing state and triplet structure conservation for some positions in miRNA duplexes. While MatureBayes employed Naive Bayes classifier, MaturePred used Support Vector Machine for the same purpose. Similarly, another tool, MiRMat [30], identifies mature miRNA duplexes in folded pre-miRNA hairpin-loop structure. However, it also considers Drosha processing site conservation as well as secondary structure free energy distribution in the duplex region of the pre-miRNA to identify mature miRNA duplexes. This tool is based on Random Forest classifier. The remaining tools to identify mature miRNA duplexes require read mapping to the genomic sequences and derived precursors structure, as already described above.

Unlike the above mentioned tools, the tool presented in this work, miReader, neither requires genomic sequences nor it is dependent upon pre-miRNA secondary structure and associated thermodynamic features. Of late, it is being argued that *de novo* secondary structure based properties may not provide the desired accuracy as they are poor discriminator [12], [31]. The present approach identifies the strength of small RNA sequencing reads information, which has in fact much lesser noise than the genomic sequences. The genome scanning based existing tools are subjected to a large number of pseudo-hairpins in genomic sequences, which could be generally low in the small RNA read data. Such read data contain only transcribed portions of genome. Also, the chances of finding a potential miRNA duplex is more in such data, as miRNAs are formed after processing of both arms of precursors. A recent pioneer wok on human miRNA profiling based on deep sequencing also concluded that in a pool of small RNA read data, a large fraction of reads belongs to miRNAs [32].

**Figure 2:**
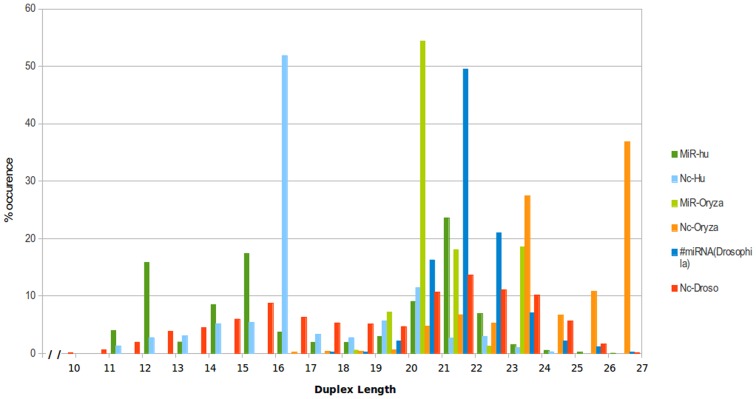
Length distribution for reads assemblies for miRNAs and non-miRNAs (ncRNAs). The assemblies from reads mapping to miRNAs had different length distributions than those observed for assemblies from reads mapping to other non-coding RNAs.

The working principle of the presented approach is also based on the fact that mature miRNA regions exhibit comparative higher degree of conservation which could be due to presence of functionally important sites in it, and interaction patterns (pairing states of nucleotides in duplex) within a mature miRNA duplex could be important for miRNA identification [1], [12], [13], [21], [22], [33], [34]. A simple test was conducted to estimate the extent to which a mature miRNA duplex could be distinguished from non-miRNA duplex, based on duplex pairing state patterns in position specific manner. The first discriminating marker was the length distribution for the assemblies or reads mapping to mature miRNAs, which were considerably different from the length distributions for assemblies for reads mapping to the non-miRNAs (**Figure 2** **)**. Based on their length, the mature miRNA and non-miRNA duplexes were clustered into different length groups and their profiles of duplex pairing states were developed. Comparisons between the profiles for mature miRNAs and non-miRNAs suggested that barring of match, for all remaining pairing states (insertion, deletion, mismatch] the profiles of non-miRNAs differed from those for miRNAs (**Supporting Material S2)**. Also, the difference between miRNA and non-miRNA profiles was not very prominent in terms of distribution of nucleotides instead of the four pairing states of duplex. Based on the above mentioned observations, it was envisaged that pairing states based properties of miRNAs could be used to identify the mature miRNAs independent of genomic sequences. Small RNA sequencing reads might be assembled in a systematic manner to discover duplexes from short read data, on which mature miRNA duplex pairing pattern could be identified. Within a mature miRNA duplex, position specific co-existence of different pairing states could work as a strong signature to identify mature miRNA duplex without any need to rely upon genomic sequences and precursor structure determination. As described, scores were obtained for all mature miRNA and non-miRNA duplexes on the basis of the 5-state transition profiles. A series of Mann-Whitney test was performed between miRNA duplexes and non-miRNA duplexes for the obtained scores. The p-values for all six species considered in this study attained highly significant values (human, *Arabidopsis*, *Oryza* and *Drosohila* (2.2 e-16); zebra fish (4.32 e-14) and *C. elegans* (2.34 e-14)), suggesting that co-existence of different pairing states in a mature miRNA duplex may work as a significant discriminatory property. So far, no significant approach had been developed to exploit the observations made in the present study for small RNA reads data, which limits miRNA information for species whose genome is yet to be sequenced. The approach presented in the current work was developed and tested for wide range of model species and delivered highly accurate results.

### Highly Reliable Performance in Absence of Genomic Sequences

The developed approach was tested for its performance over six different species viz. *Arabidopsis thaliana* (representing dicots), *Oryza sativa* (representing monocots), human (representing vertebrates system), *C. elegans* (representing nematodes), zebra fish (representing fish) and *D. melanogaster* (representing insects).

**Figure 3:**
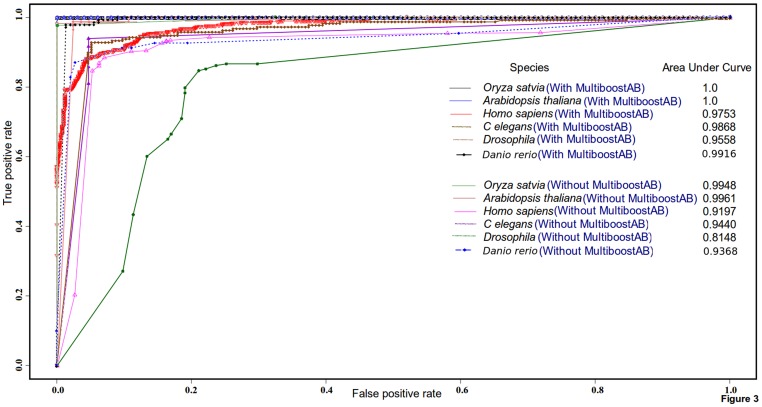
ROC plot with area under the curve values for all the six target species considered in this study. The plot suggests better and stable performance of the classifiers with multiboosting.

An *in-house* developed script was used to pick sequences randomly, and total 500 miRNAs duplexes from the positive set and 500 non-miRNAs duplexes from the negative set were drawn from the data sources for human. These duplexes were used to build the training sets. Equal amount of positive and negative instances were taken to build the test set. The associated reads for every instance were retrieved using their corresponding index. Training was done with different values of iterations going maximum up to 100 iterations for Multi-boost classifier building. For human, the developed classification system attained 90.2% accuracy with 90.4% sensitivity and 92.4% specificity for Illumina read data model. The mentioned approach attained accuracy of 92.7%, sensitivity of 92% and 93.4% specificity for human model, using read data from Roche platform. The values for Area Under the Curve (AUC) for an ROC plot and Mathew’s Correlation Coefficient (MCC) are considered among the most suitable indicators of a classifier’s performance and robustness. The observed MCC and AUC values for human were 0.8941 and 0.9753, respectively, for the training and testing sets generated from Illumina. Values for MCC and AUC were 0.912 and 0.9820, respectively, for the training and testing data generated from Roche 454 sequencing platform. Same procedure was followed for other target species with suitable input sizes, depending upon the availability of total known miRNAs. For all the target species studied here, the accuracy was always above 90% with equally high values for sensitivity, specificity, MCC and AUC. **Table 2** provides the performance details of the classification system for all target species studied here. The ROC plots for all six target species is given in **Figure 3** **,** which clearly suggests a reliable performance of the developed approach for wide range of data and species**.** For most of the target species under study, when the classifiers were tested for performance without implementing Multi-boosting, there was a significant drop in the performance of the classifiers.

**Table 2:** Performance measurement.

	*C elegans*	*Drosophila*	*Homo sapiens (Illumina)*	*Homo sapiens (Roche)*	*Arabidopsis*	*Oryza*	*Danio rerio*
Sensitivity%	97.64	93.25	90.2	92.0	100	100	97.18
Specificity%	97.64	92.13	92.4	93.4	100	100	97.93
Accuracy%	97.64	92.69	91.3	92.7	100	100	97.56
AUC	0.9868	0.9558	0.9753	0.9821	1.0	1.0	0.9916
MCC	0.8971	0.8511	0.8941	0.9120	1.0	1.0	0.9332

The presented data clearly suggests that miReader approach performed consistently with high accuracy for wide range of species.

**Figure 4:**
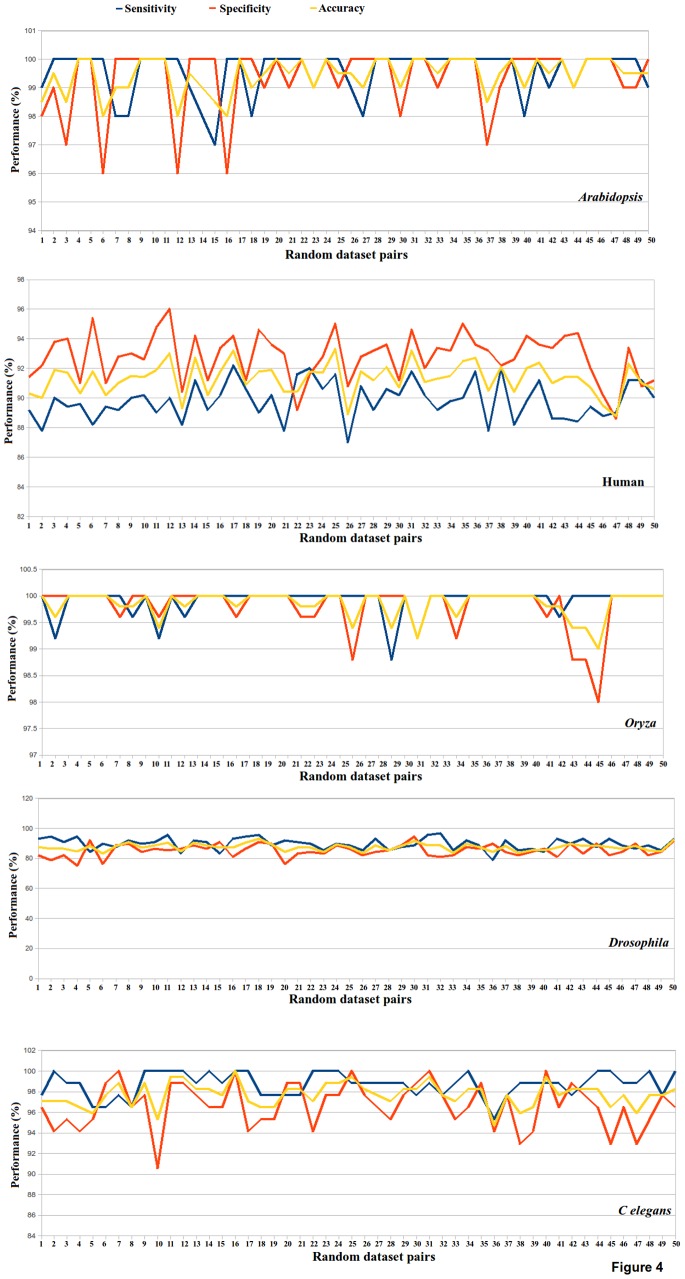
Accuracy on random datasets. Different randomly selected instances were used to build the training and testing sets every time. For all such variable tests and for all species considered, the developed models performed consistently with high accuracy.

However, sometimes the performance of some classification system models becomes limited and localized towards training dataset used. Such classifiers usually end up scoring high over a given test set, but perform poorly over new test sets. An ideal hypothesis should consistently work well for wide range of possible datasets. For verifying consistency in the observed performance for variable datasets for training and testing, total 50 pairs of random training and testing sets were generated, maintaining almost the same number of training and testing instances. The performance measured for every pair of test and training sets for these 50 tests was found to be consistent and at similar level, scoring overall accuracy of >90%. This observation was consistent for all five target species studied here. **Figure 4** shows the plots representing performance for all six target species for 50 randomized sets of training and testing dataset pairs. Also, a good amount of similarity exists between the mature miRNA regions belonging to same miRNA family. Considering this, the training and testing instances were built in a manner that no miRNA pair existed across the training and testing sets with common family. By doing so, any possibility of bias arising from similarity was curtailed. The above mentioned tests suggest a high degree of reliability of the proposed approach to identify novel miRNAs without any reference. The designed system emerged as a highly stable and consistently accurate classification system for wide range of data and species. Apart from these tests, one more test was performed to assess the degree of precision for the developed approach. A total of 126 human pre-miRNAs were taken, which could yield a non-miRNA duplex out of their stem in non overlapping manner, along with the mature miRNA duplex. In this manner, total 126 non-miRNA duplexes and 126 miRNA duplexes were obtained from these pre-miRNAs. It was ensured that none of the 126 non-miRNA duplexes overlapped with any existing miRNA duplex, as there could be more than single miRNA being formed by a given pre-miRNA sequence. The developed tool correctly identified 120 miRNA duplexes and 116 non-miRNA duplexes, suggesting a substantial precision over a highly confusing data where negative and positive duplexes co-existed in vicinity of each other. Since the developed approach works for duplex miRNAs, it may not work for conditions where complementary partner strand is not found.

Recently, it has been reported that a large number miRNA variants result due to post-transcriptional processing like trimming, addition and editing. Such miRNAs are called Iso-miRs [32]. Iso-miRs have been found to act like their miRNAs and also complement them [35]. For many, it would be a matter of interest to look for iso-miRs. Though the prime objective of this work has been to identify miRNA duplexes directly from the NGS read data, the tool has been designed in such a way that it could also be useful to identify the miRNA variants. In order to assess its performance over iso-miRs, it was tested over a read data which represented a total of 19,845 iso-miRs for ∼703 human miRNAs [35]. In order to identify potential iso-miRs, the presented tool applies a few observations made by Morin *et al* [32] as well as the data statistics observed for iso-miRs. It was observed that any group of similar reads having around 3.5% of total reads or more, mapping to any given reference miRNA could form an iso-miR group, while considering length as well as substitution/editing based grouping. Also it was observed that substitutions occurred at maximum two positions. MiReader identified 98.4% of these iso-miRs correctly. Using the above mentioned strategy, it could correctly assign the miRNA family and Iso-miR groupings for 96.8% (681 out of 703) groups. The result folder of miReader generates an iso-miR information sheet which also contains reads counts based expression measurement of miRNAs (Iso-miRs), an approach recommended by Morin *et al* [32].

### Application: Identification of Novel Mature miRNAs without Genome

**Figure 5:**
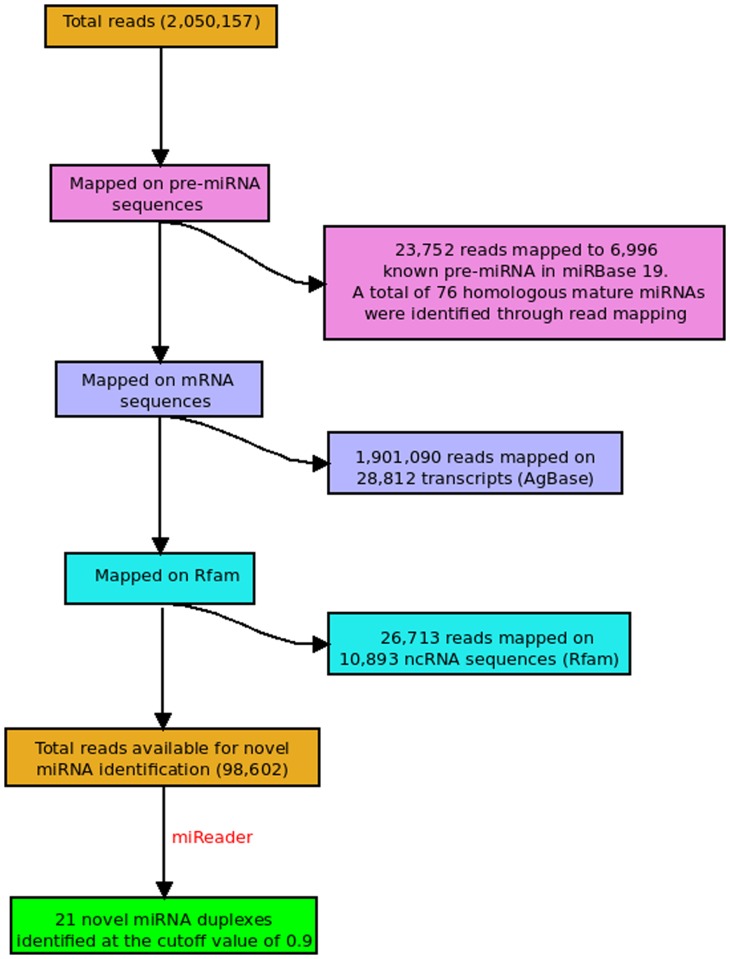
Workflow representation for novel miRNA identification in *Miscanthus*.

Encouraged by its performance over experimentally validated data for wide range of species, the presented approach was applied for identification of novel miRNAs in *Miscanthus (Miscanthus x giganteus),* a monocot grass species of family *Poaceae*. *Miscanthus* has been an important species for fuel based studies whose genome is yet not sequenced. At present there is no miRNA reported in miRBase for this species. The small RNA NGS data was made freely available for the community by the authors (GSE28755). Using miReader, the reads were assembled and screened for possible mature miRNAs. A total of 12,400,937 reads were reported from three tissues of *Miscanthus x giganteus* out of which 2,050,157 unique small RNA sequencing reads yielded 125,315 assembled candidates for processing. Perfect and imperfect complementary contigs were searched across the assembled sequences using the above mentioned approach. In this way, a total for 10,386 duplexes were found as the potential candidates, whose encoded patterns were derived and mapped into the miRNA modeling system of miReader. For the given reads data, a total of 21 duplexes qualified as novel mature miRNAs candidates in *Miscanthus x giganteus*, which also scored very high (using cut-off score of 0.9 or above) (**Supporting Material S3**). Also, prior to the assembly step and *de novo* discovery of totally novel mature miRNA candidates, the reads were screened against *Miscanthus* transcriptome sequences, non-coding RNA sequences from Rfam and miRNAs reported at miRBase v19. **Figure 5** shows the entire work flow to report the novel miRNAs in *Miscanthus*. A large number of reads mapped to known miRNAs in miRBase, suggesting presence of several homologous miRNA families in *Miscanthus*. Total of 53 different known miRNA families with 76 different known miRNAs reported in miRBase were found in *Miscanthus*. Related detail is given in **Supporting Material S4**. While performing this part of study, one more test was carried out to re-evaluate the performance of miReader over the read data from *Miscanthus*, whose data was never used to build the models of present tool. It was run against the reads data without applying any screening step against known miRNAs and non-miRNA databases. It correctly identified ∼92% of reads belonging to the known miRNAs mentioned above and correctly identified 73 out of 76 known miRNAs. Similarly, it correctly identified ∼90% of reads belonging to the non-miRNA elements mentioned above. This test supported again the reliability of miReader.

**Figure 6:**
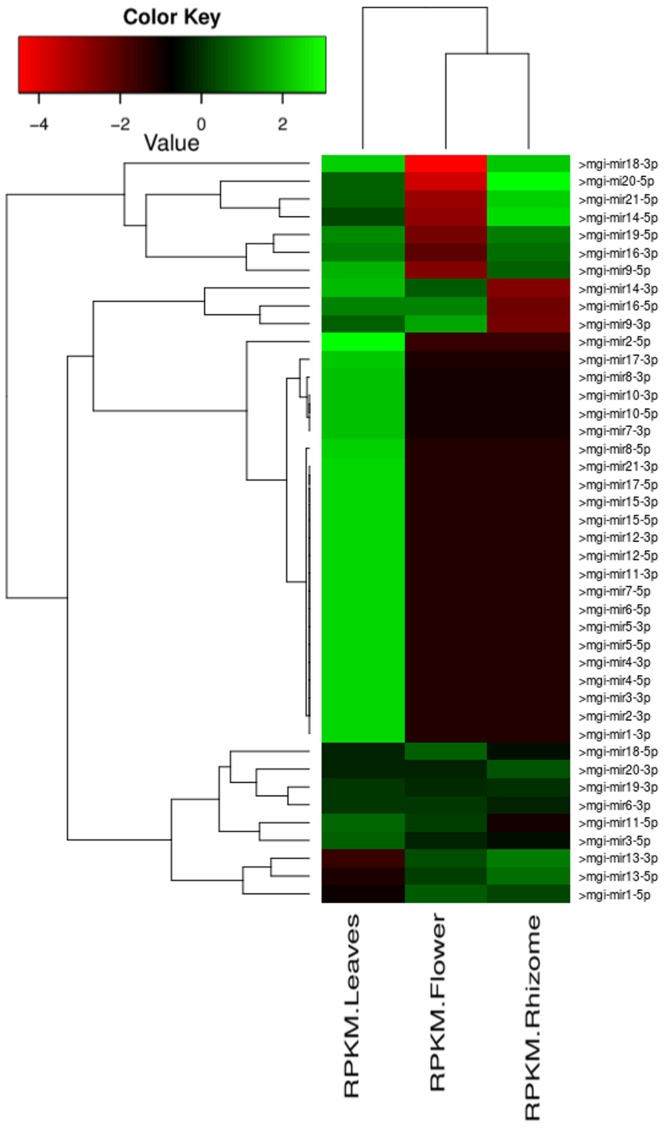
Heatmap based expression representation and clustering of identified miRNAs in *Miscanthus* **.**

Since the mentioned read data was from three different experimental condition, it was also possible to measure the normalized expressions of the identified novel miRNAs and gather further support. **Figure 6** provides the heatmap representation of identified miRNAs abundance as well as their expression based clustering, for the three experimental conditions. This also provides the first hand information about the identified miRNAs’ co-expression pattern, suggesting concerted functioning in the developmental processes by some of the identified miRNA clusters. Further, using these 21 mature miRNA candidate duplexes (yielding 42 possible mature miRNAs), whole transcriptome sequences of *Miscanthus giganteus* were scanned for miRNA targets. A reliable plant miRNA target identification system, p-TAREF [36], was applied for this part of the study which also generated confidence score of identified targets. A total of 36 target instances scored with maximum two mismatches, out of which 26 target instances had SVR score above 1.00, suggesting high possibility of the identified targets (**Supporting Material S5**).

The miRNA expression profiles vary with stage and type of tissue considered in any given study. Therefore, more the reporting of small RNA reads data is done, higher would be the ability to identify novel miRNAs. The miRNAs and targets instances reported in this study had high scores, and they have been supported with read data. Further experimental studies with the reported instances would be helpful for understanding the biology of *Miscanthus* and its regulatory systems. Application of the developed approach over the reads data from *Miscanthus* also clearly demonstrated the way it could be used to unravel the miRNAs of species whose genome is not sequenced.

### Parallel Implementation and Availability

**Figure 7:**
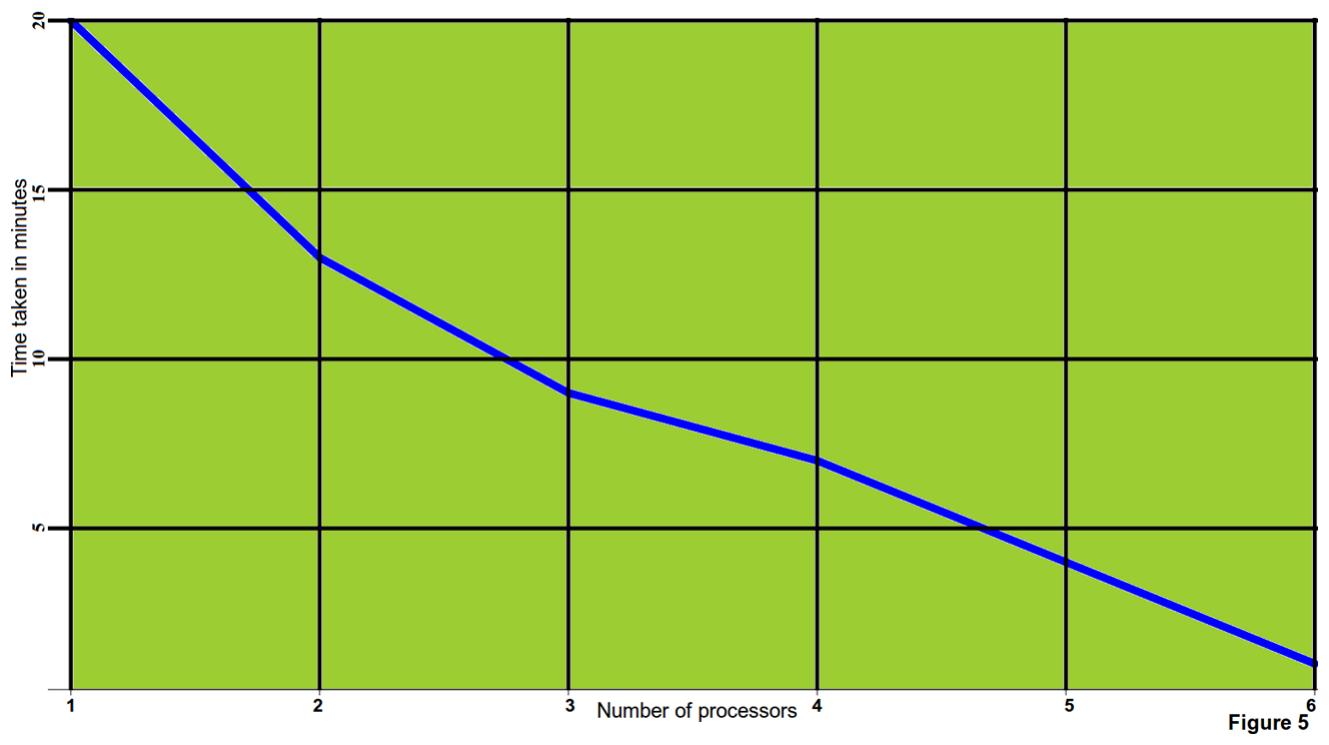
Impact of concurrency. The plot clearly shows that with concurrency the rate of execution could be enhanced several times.

The entire algorithm has been implemented as a concurrently coded tool, miReader, available for Linux as well as Windows systems in standalone GUI form. The entire implementation has been done in Java and Qt C++ library. Since any high throughput analysis demands a large amount of computing, and thus execution speed becomes a concern in most of the genome wide studies. Therefore, it becomes a pragmatic choice to develop applications with parallel architecture. In the present work, parallel coding was done using Java multi-threading library. On a given dataset, it was observed that parallel coded version drastically reduced the execution time (**Figure 7**). miReader is capable of running on multiple processors simultaneously. It utilizes Java concurrent library (JCL) to distribute multiple sequences across a given number of processors. The parallelism of miReader was implemented at two stages: A) During assembling the overlapping reads to produce assemblies and B) During complementarity search and duplex formation. To assemble the overlapping reads, miReader uses KMP algorithm which takes a linear time to search the string O(m+n) where ‘*m*’ and ‘*n*’ are the lengths of related strings. In miReader, this algorithm has been modified to search multiple strings concurrently. Using the parallel version of KMP algorithm, the time taken for assembling reduced proportionally to the number of processors provided. The process to search the most suitable duplex required global alignment of complementary sequences, which is another time consuming step with quadratic operation time. Implementation of parallelism at this step also contributed to sharp reduction in computation time. **Supporting Material S6** contains instructions to use miReader. miReader along with its documentation is freely available at: http://scbb.ihbt.res.in/2810-12/miReader.php as well as at an open access portal, Sourceforge (http://sourceforge.net/projects/mireader/).

### Conclusion

miRNAs have emerged among the central components of regulatory systems. Several computational approaches have been developed to identify precursors of miRNAs as well as locate mature miRNAs within them. However, all these methods rely upon the availability of genomic reference sequences to detect miRNAs, restricting miRNA information to only those species whose genomic sequences are available. In the present work, for the first time, an approach and a tool has been presented which could detect the mature miRNAs directly from next generation sequencing read data, without any need of reference/genomic sequences. The approach was tested over wide range of species, and the presented approach achieved high accuracy for all the target species. Using the same approach, 21 novel mature miRNA duplex candidates were identified for a plant species whose genome has not been sequenced yet and there is negligible miRNA data reported for this species in miRBase. This has clearly demonstrated clearly that in spite of unavailability of genomic sequences, the presented tool, miReader, could accurately identify the mature miRNAs directly from small RNA sequencing data. As already shown in this work that due to unavailability of genomic sequences, the current miRNA information got skewed towards the organisms with sequenced genome. It is expected that with this tool such information skew would be offset to a large extent and miRNA biology of several new species would now be possible even without availability of their genomic sequences.

## Supporting Information

Supporting Material S1 **Known miRNA distribution with respect to sequenced genomes.**
(DOC)

Supporting Material S2 **Representing the distribution profiles of various pairing states in miRNA and non-miRNA duplexes for different lengths and species.**
(PDF)

Supporting Material S3 **Identified miRNAs in** ***Miscanthus*** **, along with their expression values.**
(DOC)

Supporting Material S4 **Known homologous miRNAs found in** ***Miscanthus*** **.**
(DOC)

Supporting Material S5 **Identified targets for novel miRNAs in** ***Miscanthus.***
(DOC)

Supporting Material S6 **miReader application instructions.**
(DOC)
