# Supplementary material for: miReader: Discovering Novel miRNAs in Species without Sequenced Genome
Source: PLoS One. 2013 Jun 21;8(6):e66857. doi: 10.1371/journal.pone.0066857 (PMC3689854; doi:10.1371/journal.pone.0066857)
Supplement: Supporting Material S1 — Known miRNA distribution with respect to sequenced genomes. (DOC) [file pone.0066857.s001.doc]

Supplementary Table 1: Known miRNA distribution with respect to sequenced genomes.

TableA: Total number of reported miRNAs in miRBase for species whose genome is sequenced.

| *Species* | Number of miRNAs reported in miRBase |
| --- | --- |
| *Aedes aegypti* | 101 |
| *Anolis carolinensis* | 282 |
| *Anopheles gambiae* | 67 |
| *Apis mellifera* | 218 |
| *Acyrthosiphon pisum* | 117 |
| *Arabidopsis thaliana* | 299 |
| *Brachypodium distachyon* | 135 |
| *Branchiostoma floridae* | 156 |
| *Bombyx mori* | 489 |
| *Brassica napus* | 90 |
| *Bos taurus* | 766 |
| *Caenorhabditis briggsae* | 140 |
| *Caenorhabditis elegans* | 223 |
| *Canis familiaris* | 323 |
| *Ciona intestinalis* | 348 |
| *Culex quinquefasciatus* | 74 |
| *Chlamydomonas reinhardtii* | 50 |
| *Caenorhabditis remanei* | 109 |
| *Ciona savignyi* | 27 |
| *Capitella teleta* | 124 |
| *Drosophila ananassae* | 76 |
| *Drosophila erecta* | 81 |
| *Drosophila grimshawi* | 82 |
| *Drosophila melanogaster* | 238 |
| *Drosophila mojavensis* | 71 |
| *Drosophila persimilis* | 75 |
| *Drosophila pseudoobscura* | 210 |
| *Daphnia pulex* | 44 |
| *Danio rerio* | 344 |
| *Drosophila sechellia* | 78 |
| *Drosophila simulans* | 136 |
| *Drosophila virilis* | 74 |
| *Drosophila willistoni* | 77 |
| *Drosophila yakuba* | 80 |
| *Epstein Barr virus* | 25 |
| *Equus caballus* | 341 |
| *Echinococcus granulosus* | 23 |
| *Echinococcus multilocularis* | 22 |
| *Fugu rubripes* | 129 |
| *Gallus gallus* | 684 |
| *Gorilla gorilla* | 322 |
| *Glycine max* | 506 |
| *Heliconius melpomene* | 92 |
| *Homo sapiens* | 1600 |
| *Ixodes scapularis* | 49 |
| *Kaposi sarcoma-associated herpesvirus* | 13 |
| *Lottia gigantea* | 60 |
| *Malus domestica* | 206 |
| *Monodelphis domestica* | 156 |
| *Mareks disease virus* | 14 |
| *Macaca mulatta* | 535 |
| *Mus musculus* | 855 |
| *Medicago truncatula* | 675 |
| *Nematostella vectensis* | 49 |
| *Nasonia vitripennis* | 53 |
| *Ornithorhynchus anatinus* | 337 |
| *Oryzias latipes* | 168 |
| *Oryza sativa* | 591 |
| *Pristionchus pacificus* | 124 |
| *Physcomitrella patens* | 229 |
| *Pongo pygmaeus* | 633 |
| *Populus trichocarpa* | 323 |
| *Pan troglodytes* | 655 |
| *Ricinus communis* | 63 |
| *Rattus norvegicus* | 446 |
| *Sorghum bicolor* | 206 |
| *Sarcophilus harrisii* | 67 |
| *Schistosoma japonicum* | 55 |
| *Solanum lycopersicum* | 44 |
| *Schistosoma mansoni* | 20 |
| *Schmidtea mediterranea* | 148 |
| *Strongylocentrotus purpuratus* | 62 |
| *Sus scrofa* | 271 |
| *Triticum aestivum* | 42 |
| *Tribolium castaneum* | 220 |
| *Taeniopygia guttata* | 243 |
| *Tetraodon nigroviridis* | 132 |
| *Vitis vinifera* | 163 |
| *Xenopus tropicalis* | 189 |
| *Zea mays* | 172 |

TableB: Total known miRNAs reported in miRBase for species whose genome is not sequenced.

| *Species* | Number of miRNAs reported in miRBase |
| --- | --- |
| *Acacia auriculiformis* | 7 |
| *Ateles geoffroyi* | 60 |
| *Arachis hypogaea* | 23 |
| *Arabidopsis lyrata* | 201 |
| *Acacia mangium* | 3 |
| *Aquilegia caerulea* | 45 |
| *Ascaris suum* | 97 |
| *Aegilops tauschii* | 2 |
| *Bruguiera cylindrica* | 4 |
| *Bruguiera gymnorhiza* | 4 |
| *Bovine herpesvirus 1* | 10 |
| *BK polyomavirus* | 1 |
| *Brugia malayi* | 104 |
| *Brassica oleracea* | 6 |
| *Bandicoot papillomatosis carcinomatosis virus type 1* | 1 |
| *Bandicoot papillomatosis carcinomatosis virus type 2* | 1 |
| *Brassica rapa* | 39 |
| *Cynara cardunculus* | 48 |
| *Citrus clementine* | 5 |
| *Cyprinus carpio* | 134 |
| *Cricetulus griseus* | 200 |
| *Cerebratulus lacteus* | 2 |
| *Cucumis melo* | 120 |
| *Carica papaya* | 1 |
| *Citrus reticulata* | 4 |
| *Citrus sinensis* | 60 |
| *Citrus trifoliata* | 6 |
| *Digitalis purpurea* | 13 |
| *Elaeis guineensis* | 6 |
| *Ectocarpus siliculosus* | 26 |
| *Festuca arundinacea* | 15 |
| *Gossypium herbaceum* | 1 |
| *Gossypium hirsutum* | 37 |
| *Glottidia pyramidata* | 1 |
| *Gossypium raimondii* | 4 |
| *Glycine soja* | 13 |
| *Helianthus annuus* | 8 |
| *Helianthus argophyllus* | 3 |
| *Hevea brasiliensis* | 28 |
| *Helianthus ciliaris* | 3 |
| *Haemonchus contortus* | 187 |
| *Hippoglossus hippoglossus* | 1 |
| *Human herpesvirus 6B* | 4 |
| *Hydra magnipapillata* | 17 |
| *Helianthus paradoxus* | 3 |
| *Helianthus petiolaris* | 3 |
| *Haliotis rufescens* | 5 |
| *Herpes Simplex Virus 1* | 17 |
| *Herpes Simplex Virus 2* | 18 |
| *Helianthus tuberosus* | 16 |
| *Herpesvirus of turkeys* | 17 |
| *Hordeum vulgare* | 67 |
| *JC polyomavirus* | 1 |
| *Lemur catta* | 16 |
| *Lotus japonicus* | 3 |
| *Lagothrix lagotricha* | 48 |
| *Locusta migratoria* | 7 |
| *Mouse cytomegalovirus* | 18 |
| *Manihot esculenta* | 10 |
| *Macropus eugenii* | 3 |
| *Macaca nemestrina* | 74 |
| *Manduca sexta* | 95 |
| *Nasonia giraulti* | 32 |
| *Nasonia longicornis* | 28 |
| *Nicotiana tabacum* | 163 |
| *Ovis aries* | 55 |
| *Oikopleura dioica* | 66 |
| *Picea abies* | 40 |
| *Pygathrix bieti* | 11 |
| *Pinus densata* | 31 |
| *Populus euphratica* | 5 |
| *Petromyzon marinus* | 244 |
| *Paralichthys olivaceus* | 20 |
| *Pan paniscus* | 88 |
| *Pseudorabies virus* | 13 |
| *Pinus taeda* | 37 |
| *Phaseolus vulgaris* | 8 |
| *Rehmannia glutinosa* | 13 |
| *Rhesus lymphocryptovirus* | 36 |
| *Rhipicephalus microplus* | 24 |
| *Saccoglossus kowalevskii* | 91 |
| *Saguinus labiatus* | 42 |
| *Selaginella moellendorffii* | 58 |
| *Strigamia maritima* | 3 |
| *Saccharum officinarum* | 16 |
| *Salvia sclarea* | 18 |
| *Saccharum ssp.* | 18 |
| *Symphalangus syndactylus* | 11 |
| *Solanum tuberosum* | 11 |
| *Theobroma cacao* | 82 |
| *Terebratulina retusa* | 1 |
| *Triticum turgidum* | 1 |
| *Tetranychus urticae* | 52 |
| *Vigna unguiculata* | 18 |
| *Xenoturbella bocki* | 8 |
| *Xenopus laevis* | 22 |
