# Supplementary material for: miReader: Discovering Novel miRNAs in Species without Sequenced Genome
Source: PLoS One. 2013 Jun 21;8(6):e66857. doi: 10.1371/journal.pone.0066857 (PMC3689854; doi:10.1371/journal.pone.0066857)
Supplement: Supporting Material S2 — Representing the distribution profiles of various pairing states in miRNA and non-miRNA duplexes for different lengths and species. (PDF) [file pone.0066857.s002.pdf]

Length 20

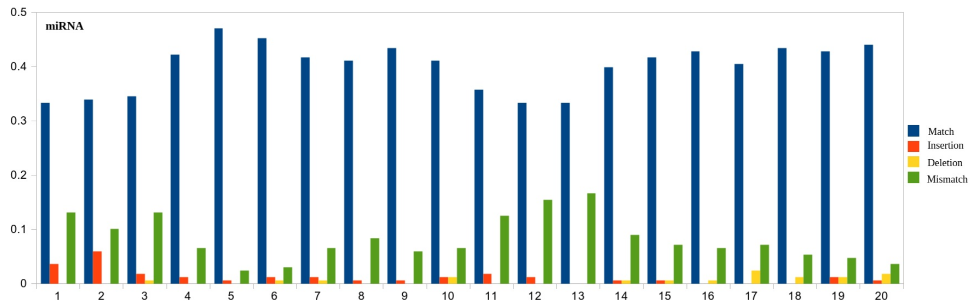

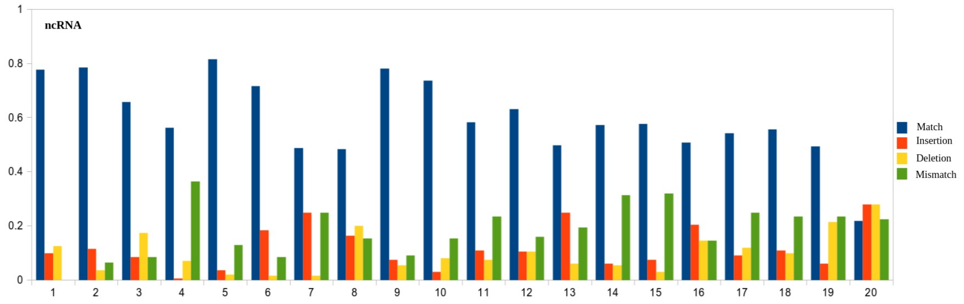

Length 21

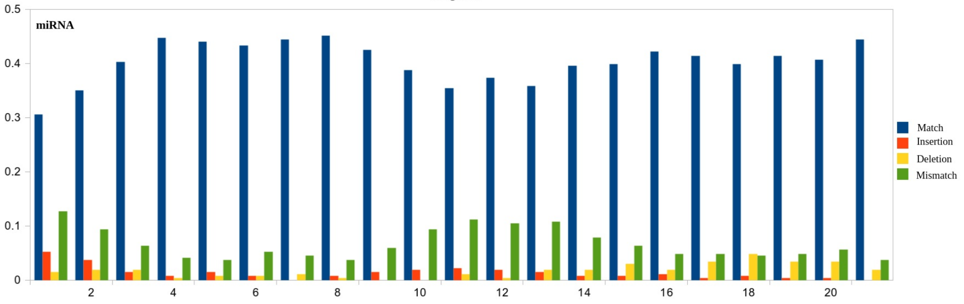

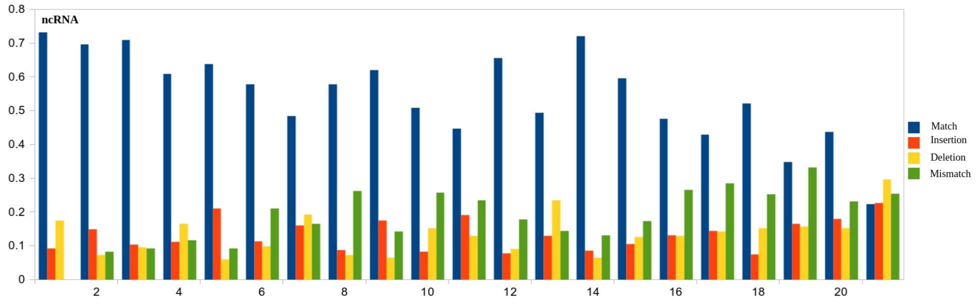

Length 22

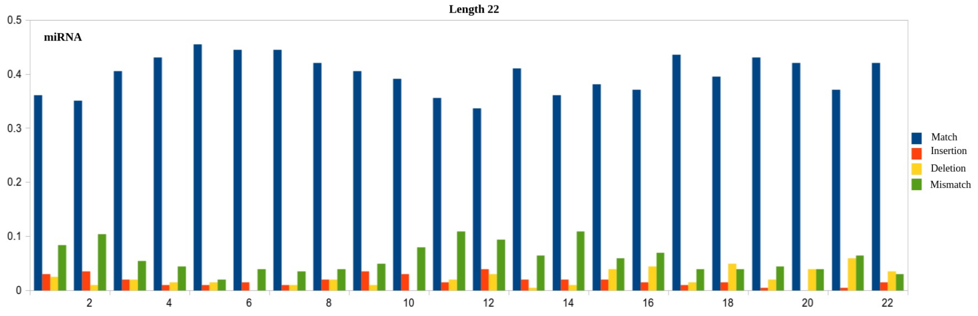

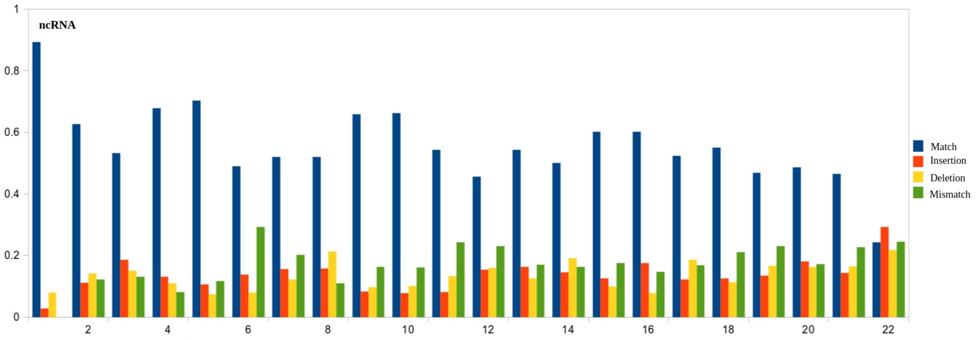

S-Figure2(C): Duplex pairing state distributions for different positions in miRNA and non-miRNA duplexes of different lengths in Drosophila.

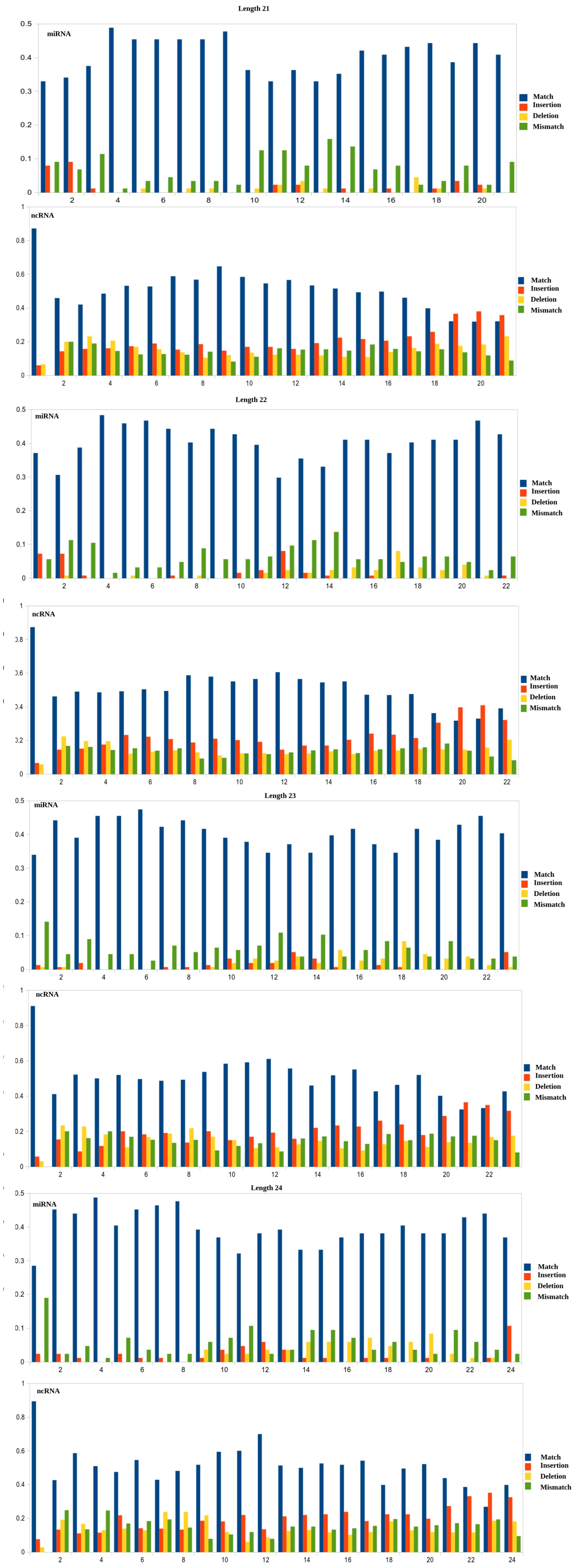

S-Figure2(B): Duplex pairing states distributions for different positins in duplex, for miRNAs and non-miRNAs of different lengths in c. elegans

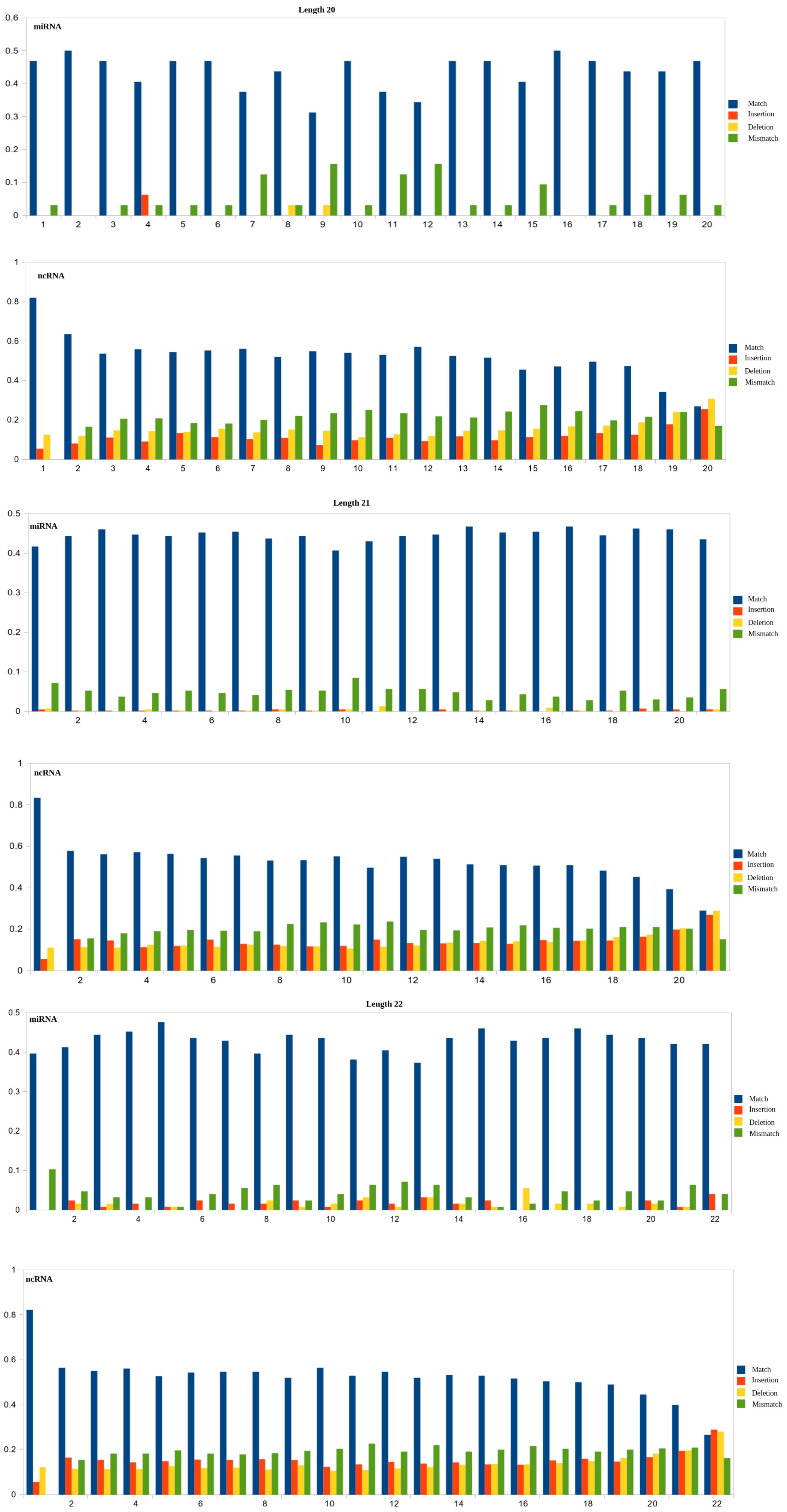

S-Figure2(A): Duplex pairing states distribution for different positions in miRNAs and non-miRNA duplexes of different lengths, from Arabidop
