# Supplementary material for: miReader: Discovering Novel miRNAs in Species without Sequenced Genome
Source: PLoS One. 2013 Jun 21;8(6):e66857. doi: 10.1371/journal.pone.0066857 (PMC3689854; doi:10.1371/journal.pone.0066857)
Supplement: Supporting Material S3 — Identified miRNAs in Miscanthus , along with their expression values. (DOC) [file pone.0066857.s003.doc]

Supplementary Table S3: Identified mature miRNAs from *Miscanthus giganteus* along with corresponding identification score and their respective expression values (in RPKM).

| miRNA id | RPKM(Leaves) | RPKM(Flower) | RPKM(Rhizome) | Score |
| --- | --- | --- | --- | --- |
| >mgi-mir1-5p | 10.853820570890123 | 33.01784125655866 | 26.17137308213561 | 0.9983644373309111 |
| >mgi-mir1-3p | 10.853820570890123 | 0.0 | 0.0 |  |
| >mgi-mir2-5p | 21.707641141780247 | 0.0 | 0.0 | 0.9983644373309111 |
| >mgi-mir2-3p | 10.853820570890123 | 0.0 | 0.0 |  |
| >mgi-mir3-5p | 21.707641141780247 | 11.005947085519555 | 8.723791027378537 | 0.9998491888672943 |
| >mgi-mir3-3p | 10.853820570890123 | 0.0 | 0.0 |  |
| >mgi-mir4-5p | 10.853820570890123 | 0.0 | 0.0 | 0.9983644373309111 |
| >mgi-mir4-3p | 10.853820570890123 | 0.0 | 0.0 |  |
| >mgi-mir5-5p | 10.853820570890123 | 0.0 | 0.0 | 0.9983644373309111 |
| >mgi-mir5-3p | 10.853820570890123 | 0.0 | 0.0 |  |
| >mgi-mir6-5p | 10.853820570890123 | 0.0 | 0.0 | 0.9983644373309111 |
| >mgi-mir6-3p | 10.853820570890123 | 11.005947085519555 | 8.723791027378537 |  |
| >mgi-mir7-5p | 10.853820570890123 | 0.0 | 0.0 | 0.999999999958995 |
| >mgi-mir7-3p | 7.893687687920089 | 0.0 | 0.0 |  |
| >mgi-mir8-5p | 10.419667748054518 | 0.0 | 0.0 | 0.997091272768767 |
| >mgi-mir8-3p | 8.140365428167591 | 0.0 | 0.0 |  |
| >mgi-mir9-5p | 21.707641141780247 | 0.0 | 8.723791027378537 | 0.997091272768767 |
| >mgi-mir9-3p | 7.893687687920089 | 16.00865030621026 | 0.0 |  |
| >mgi-mir10-5p | 7.893687687920089 | 0.0 | 0.0 | 0.543119331339669 |
| >mgi-mir10-3p | 7.893687687920089 | 0.0 | 0.0 |  |
| >mgi-mir11-5p | 32.561461712670365 | 22.01189417103911 | 8.723791027378537 | 0.999999999958995 |
| >mgi-mir11-3p | 10.853820570890123 | 0.0 | 0.0 |  |
| >mgi-mir12-5p | 10.853820570890123 | 0.0 | 0.0 | 0.543119331339669 |
| >mgi-mir12-3p | 10.853820570890123 | 0.0 | 0.0 |  |
| >mgi-mir13-5p | 54.269102854450615 | 132.07136502623464 | 218.0947756844634 | 0.543119331339669 |
| >mgi-mir13-3p | 10.853820570890123 | 44.02378834207822 | 69.7903282190283 |  |
| >mgi-mir14-5p | 7.893687687920089 | 0.0 | 38.067451755833616 | 0.543119331339669 |
| >mgi-mir14-3p | 23.681063063760266 | 8.00432515310513 | 0.0 |  |
| >mgi-mir15-5p | 10.853820570890123 | 0.0 | 0.0 | 0.997091272768767 |
| >mgi-mir15-3p | 10.853820570890123 | 0.0 | 0.0 |  |
| >mgi-mir16-5p | 10.018911296206268 | 10.15933577124882 | 0.0 | 0.997091272768767 |
| >mgi-mir16-3p | 7.893687687920089 | 0.0 | 6.344575292638935 |  |
| >mgi-mir17-5p | 10.853820570890123 | 0.0 | 0.0 | 0.543119331339669 |
| >mgi-mir17-3p | 8.982472196598723 | 0.0 | 0.0 |  |
| >mgi-mir18-5p | 10.853820570890123 | 22.01189417103911 | 8.723791027378537 | 0.997091272768767 |
| >mgi-mir18-3p | 109.23845219734575 | 0.0 | 101.30854096310559 |  |
| >mgi-mir19-5p | 12.40436636673157 | 0.0 | 9.970046888432613 | 0.997091272768767 |
| >mgi-mir19-3p | 1064.6182264316571 | 918.7573219216324 | 983.1333192593551 |  |
| >mgi-mi20-5p | 21.707641141780247 | 0.0 | 113.40928335592098 | 0.9983644373309111 |
| >mgi-mir20-3p | 2564.8412918288045 | 2621.1086289821956 | 4211.577883679053 |  |
| >mgi-mir21-5p | 10.853820570890123 | 0.0 | 34.89516410951415 | 0.997091272768767 |
| >mgi-mir21-3p | 10.853820570890123 | 0.0 | 0.0 |  |
