# Supplementary material for: miReader: Discovering Novel miRNAs in Species without Sequenced Genome
Source: PLoS One. 2013 Jun 21;8(6):e66857. doi: 10.1371/journal.pone.0066857 (PMC3689854; doi:10.1371/journal.pone.0066857)
Supplement: Supporting Material S4 — Known homologous miRNAs found in Miscanthus . (DOC) [file pone.0066857.s004.doc]

Supplementary Data S4: List of mature mIRNAs identified through read mapping to known miRNAs reported in miRBase 19

miR100

miR1134

miR1192

miR1286

miR1310

miR1362

miR1421m

miR1520f

miR156a

miR156b

miR156c

miR156d

miR156e

miR156f

miR156g

miR156h

miR156j

miR156k

miR156p

miR156t

miR156u

miR156v

miR156w

miR184

miR1877

miR1895

miR1906

miR1940

miR204

miR2096

miR263a

miR2863c

miR2911

miR2962

miR3254

miR3257

miR3416

miR3605

miR3835

miR3940

miR398a

miR408b

miR4346

miR4371a

miR4391

miR4398

miR4556

miR4577

miR4644

miR468

miR4773

miR4952

miR5021

miR5198

miR529

miR529a

miR529b

miR529c

miR529d

miR529e

miR529f

miR529g

miR5300

miR5544

miR5557

miR5718

miR574

miR6214

miR6219

miR6370

miR638

miR658

miRH5

miRI5

miRM21

miRM95

Figure: Species wise distribution of homologous miRNAs detected in *Miscanthus*
