# Supplementary material for: miReader: Discovering Novel miRNAs in Species without Sequenced Genome
Source: PLoS One. 2013 Jun 21;8(6):e66857. doi: 10.1371/journal.pone.0066857 (PMC3689854; doi:10.1371/journal.pone.0066857)
Supplement: Supporting Material S5 — Identified targets for novel miRNAs in Miscanthus. (DOC) [file pone.0066857.s005.doc]

Supplementary Table S3. Identified targets of Miscanthus x giganteus transcriptome by p-TAREF.

| Libraray mIRNA | Library pattern | Transcript | MiRNA id | Target sequence | Mismatches | Predicted pattern | Start | End | SVR score |
| --- | --- | --- | --- | --- | --- | --- | --- | --- | --- |
| ath-mir169g-3p | XXXXXMXXMMMXWXMXXXXXX | ATLocus_4_Transcript_1/1_Confidence_1.000 | mgi-mir13-5p | GCAGGGGAGCAAGCCCCUGUGUGAUUUCU | 2 | MbbbbbWXXMXXXXXMXXMMMXXMXXXXX | 745 | 774 | 1.01303 |
| ath-mir844 | XXXXMXXWXXXXXMWXMXXWX | ATLocus_8_Transcript_1/1_Confidence_1.000 | mgi-mir8-3p | GAGCGGAAGGCCGCCGGGGAAGGCAA | 2 | WMXMMXXXXMXXWXXXXXWMXXWXXX | 836 | 862 | 1.76811 |
| ath-mir172b-3p | XXMXMXMXMXMMXXXMXMXM | ATLocus_8_Transcript_1/1_Confidence_1.000 | mgi-mir20-3p | CCGACCGCUUCCUCCCGGGAGGCUCGCA | 2 | XXWXXMXXMXMXMXXXWMXXXMXMXMbb | 1314 | 1342 | 1.82428 |
| ath-mir393a | XXMXXMXXXXXXXMMWMMXMXX | ATLocus_14_Transcript_1/1_Confidence_1.000 | mgi-mir21-3p | CGCCUCCUCCAGCUUCUGCUGCUG | 2 | XMXXMXXMXXXXXXMMWMXMXXMX | 25 | 49 | 1.9845 |
| ath-mir393b | XXMXXMXXXXXXXMMWMMXMXX | ATLocus_14_Transcript_1/1_Confidence_1.000 | mgi-mir21-3p | CGCCUCCUCCAGCUUCUGCUGCUG | 2 | XMXXMXXMXXXXXXMMWMXMXXMX | 25 | 49 | 1.9845 |
| ath-mir844 | XXXXMXXWXXXXXMWXMXXWX | ATLocus_20_Transcript_1/1_Confidence_1.000 | mgi-mir2-3p | NGGAAAAGAAGAACAGAUUGAUCU | 2 | XWXXXXXMXXWXXXXXMMXMXXXM | 590 | 613 | 1.46787 |
| ath-mir844 | XXXXMXXWXXXXXMWXMXXWX | ATLocus_28_Transcript_1/1_Confidence_1.000 | mgi-mir2-3p | CGCAGAAGGAGAAGGGGGGAUCGA | 2 | XWMXXXXMXXWXXXXXWXMXMXWX | 120 | 144 | 2.54694 |
| ath-mir172b-3p | XMMXMXMXMXMMXXXMXMXM | ATLocus_31_Transcript_1/1_Confidence_1.000 | mgi-mir5-3p | AUGGGAGAGGCCUCGCCGUCGAUC- | 2 | XbMXXMWXMXMXMXMXXXMXMXMXB | 95 | 119 | 1.76095 |
| ath-mir172b-3p | XXMXMXMXMXMMXXXMXMXM | ATLocus_31_Transcript_1/1_Confidence_1.000 | mgi-mir5-3p | AUGGGAGAGGCCUCGCCGUCGAUC- | 2 | XbMXXMWXMXMXMXMXXXMXMXMXB | 95 | 119 | 1.76095 |
| ath-mir172b-3p | XXMXMXMXMXMMXXXMXMXM | ATLocus_52_Transcript_1/1_Confidence_1.000 | mgi-mir5-5p | AGAUCGAGAUGUACUCGCCGGCCU | 2 | XXMXXXMXMXMXMMXMMXXXMXMX | 280 | 304 | 1.14264 |
| ath-mir844-3p | XXXXXXXWXXMMXXWXMXXXX | ATLocus_54_Transcript_1/1_Confidence_1.000 | mgi-mir19-3p | CCUCGACGUCGUCACCACAAGC- | 2 | XXXXXMXWXXMMXXXXMXXXXMB | 205 | 227 | 2.0084 |
| ath-mir172b-3p | XMMXMXMXMXMMXXXMXMXM | ATLocus_57_Transcript_1/1_Confidence_1.000 | mgi-mir16-5p | CUGGCUCCCCGCAUCACAUCGCAAUCG-C | 2 | bbbWXMMXXXMMXMXMXXMMXXMXMXMBM | 5 | 33 | 1.64242 |
| ath-mir169g-3p | XXXXXMXXMMMXWXMXXXXXX | ATLocus_100_Transcript_1/1_Confidence_1.000 | mgi-mir21-3p | UGCCGGUGCCGCGGGCGUAGGCGAGGC | 2 | MMbbbMMXXXXXMXXXMMXXMXXXXXX | 85 | 112 | 1.82254 |
| ath-mir844-3p | XXXXXXXWXXMMXXWXMXXXW | ATLocus_116_Transcript_1/1_Confidence_1.000 | mgi-mir19-5p | CACCGGCGGCUACCGCGUCUG | 1 | XXXXXXMWXXMMXXXMXXXXW | 560 | 581 | 1.51049 |
| ath-mir172b-3p | XMMXMXMXMXMMXXXMXMXM | ATLocus_123_Transcript_1/1_Confidence_1.000 | mgi-mir2-5p | N-GAUGAGCAAGCGCACGGCAUC- | 2 | XBMXXMMXXMXMXMXXXMXMXMXB | 41 | 62 | 2.13992 |
| ath-mir169g-3p | XXXXXMXXMMMXWXMXXXXXX | ATLocus_126_Transcript_1/1_Confidence_1.000 | mgi-mir11-3p | AACGGUAUCCUGAACGUGAAGGCG | 2 | MXXXXXXXXMXMMMXWMXXXXXXM | 447 | 471 | 1.39366 |
| ath-mir169g-3p | XXXXXMXXMMMXWXMXXXXXX | ATLocus_126_Transcript_1/1_Confidence_1.000 | mgi-mir12-3p | AACGGUAUCCUGAACGUGAAGGCG | 2 | MXXXXXXXXMXMMMXWMXXXXXXM | 447 | 471 | 1.39366 |
| ath-mir172b-3p | XXMXMXMXMXMMXXXMXMXM | ATLocus_127_Transcript_1/1_Confidence_1.000 | mgi-mir7-5p | ACGCUGCGACCGGGAUGGAUCUCA | 2 | XMMXXMXMXMXXMMXXXXMXMXMM | 158 | 182 | 1.02508 |

Identified positive miRNAs

>mgi-mir1-5p

GAUUUACUGACGGAUUCUAUAAUU

>mgi-mir1-3p

UUAAAUGGAUGAUCAAGGUAUUAA

>mgi-mir2-5p

AAUGCCGUCGGUGUAGUAGACGUC

>mgi-mir2-3p

UAAUGGUACCUGUAGUGUUUGCAU

>mgi-mir3-5p

GACAAAUAACAACGAAAACUGCUA

>mgi-mir3-3p

UUGUUUAUGGUAGAAUUUGAUGAG

>mgi-mir4-5p

AUACAAGUAAAAGACUCAUUGACC

>mgi-mir4-3p

UCUGUUUGGUUUUUCAGUAGCUGU

>mgi-mir5-5p

ACGGCAAGCACAAGAAUAUAGAUC

>mgi-mir5-3p

UAUCGUUUUUGUUUCUGUAACCGG

>mgi-mir6-5p

UCAAGACUGAAGCCAUGGUAUGGA

>mgi-mir6-3p

AUUUCUGAAUUUGGCACUAUGCAU

>mgi-mir7-5p

ACGCGACCACGGCACGGUGAAGCU

>mgi-mir7-3p

UUUGUUGGAGCUGGACUGUUUUGCCUCCGAUUG

>mgi-mir8-5p

AAAAGGGUCACAUCAAAUCUUACGG

>mgi-mir8-3p

UUUUUUCACUGUAUAUUGGCUUGUAUUGUGUC

>mgi-mir9-5p

AAUGAACUCGUAAAAUAUAGAAGA

>mgi-mir9-3p

UUAUUUGGAUGUUGCAUAUUUUUGUCUUAUUCA

>mgi-mir10-5p

GUUGUCGUGGCGGGGCCGAGAGGGGCGGAUGGC

>mgi-mir10-3p

UUGCAGCGGCGGCGCGGCUCUCACCGCAUGCCC

>mgi-mir11-5p

UAAAAUAGGCUCUAGUCCCGGCAU

>mgi-mir11-3p

GUUUUGUUAGAAGACGGGGUUGUA

>mgi-mir12-5p

CAAAAUAAGCUCUAGUCCCGGUAU

>mgi-mir12-3p

GUUUUGUUAGAAGACGGGGUUGUA

>mgi-mir13-5p

GAAAGAUGGUGAUCUAUGCCUGAG

>mgi-mir13-3p

CUUUCUGUAAUCGAAUACGAGCUU

>mgi-mir14-5p

UUCAGUUGACAUGGCCUGGUUCAGUCAUAUUAC

>mgi-mir14-3p

AAGUUAGUCGUCUAGGGGUUAGUCGGUAUGCUG

>mgi-mir15-5p

AUUUGGGUCCACCACUACAUAUGC

>mgi-mir15-3p

UGGACUUGCGUGGAAGCGUGUAUG

>mgi-mir16-5p

CUGAUCAACCUGUAUUGCGGUGCUAA

>mgi-mir16-3p

GGCUGGUUCGGCACUGUGCGGCUGUCGUGUUGG

>mgi-mir17-5p

AAUGAACUAGAAGUAAGAAAAAAU

>mgi-mir17-3p

UUGUUUGACUUGUUUUUUUUUUUUUUUUU

>mgi-mir18-5p

AUCUUCAUAAGUUUGACUGGCAUU

>mgi-mir18-3p

UAGGAGUAGUUAGCAUGGCUGUGGUGUUCCG

>mgi-mir19-5p

AGAACCUAUAUGCACUCUGGU

>mgi-mir19-3p

UCUUGGAUUUAUGAAAGACUGAG

>mgi-mir20-5p

UGGACGGAAUGACUUGUACGGCAA

>mgi-mir20-3p

GUUUGUUUGAUGGUACGUGUCGUAUG

>mgi-mir21-5p

AAAGACUGCGCAGAAGACCAACAG

>mgi-mir21-3p

UUUUUGGCCUGUUCGUUGGUUGGU
