# Supplementary material for: miReader: Discovering Novel miRNAs in Species without Sequenced Genome
Source: PLoS One. 2013 Jun 21;8(6):e66857. doi: 10.1371/journal.pone.0066857 (PMC3689854; doi:10.1371/journal.pone.0066857)
Supplement: Supporting Material S6 — miReader application instructions. (DOC) [file pone.0066857.s006.doc]

**How to use miReader**

*Linux:*

Dependencies:

1. Java 5 or greater.
2. QT4 (for GUI)
3. Any Linux distribution (x86_64)

Run miReader:
Graphical user interface (GUI)

Extract or unzip the miReader_Linux.tar.gz file, to run miReader execute ./install (need not to be root) this file will create a binary named miReader. Double click on this binary will show a splash screen and after few seconds will show user interface. Select input file, output folder and number of processors, choose model organism out of 5 organism by clicking on radiobutton.

Command line interface (CLI)

Extract miReader_Linux.tar.gz and execute “java -jar miReader_dicot.jar inputfile (can be in fasta format or in fastq format) output_folder_destination (/home/user/...) number_of_processors” (without quotes). As miReader does not require any third party dependencies it can be (theoretically) run on any system with Linux O.S having Java version > 5.

*Windows:*

Dependencies:

1. Java 5 or greater.
2. Windows (x86_64)

Run miReader:

Graphical user interface (GUI)

Extract the miReader_windows.rar file double click on miReader and follow the above steps to choose input file output folder destination, number of processor and model species.

Command line interface (CLI)

Follow the above steps described to run CLI version of miReader in Linux.

Note to run miReader for different model species in above command replace the jar file with respective model

1. miReader_dicot.jar : model - Arabidopsis thaliana
2. miReader_monocot.jar : model - Oryza satvia
3. miReader_human.jar : model - Homo sapiens
4. miReader_nematod.jar : model - Caenorhabditis elegans
5. miReader_fly.jar : model - Drosophila melanogaster

For example run java -jar miReader_dicot.jar inputfile output_folder number_of_processors if you want to use dicot (*Arabidopsis*) as model.

Or java -jar miReader_human.jar inputfile output_folder number of processors to identify reads for human model.


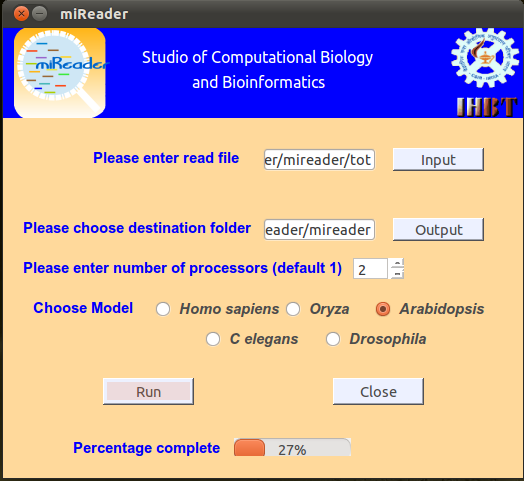
Figure 1: Snapshot of miReader GUI
